# Supplementary material for: Immunological Study of Combined Administration of SARS-CoV-2 DNA Vaccine and Inactivated Vaccine
Source: Vaccines (Basel). 2022 Jun 10;10(6):929. doi: 10.3390/vaccines10060929 (PMC9228235; doi:10.3390/vaccines10060929)
Supplement: Supplementary file 1 [file vaccines-10-00929-s001.zip › vaccines-1745160-supplementary.pdf]

vaccines

MDPI

Type of the Paper (Article)

Immunological study of combined administration of SARS-CoV-2 DNA vaccine and inactivated vaccine

Ziyan Meng, Danjing Ma, Suqin Duan, JingJing Zhang, Rong Yue, Xinghang Li, Yang Gao, Xueqi Li, Fengyuan Zeng, Xiangxiong Xu, Guorun Jiang, Yun Liao, Shengtao Fan, Zhenye Niu, Dandan Li, Li Yu, Heng Zhao, XingLi Xu, Lichun Wang, Ying Zhang, Longding Liu*, Qihan Li*

Institute of Medical Biology, Chinese Academy of Medical Sciences & Peking Union Medical College, Yunnan Key laboratory of Vaccine Research and Development on Severe Infectious Diseases, Kunming 650118, China.

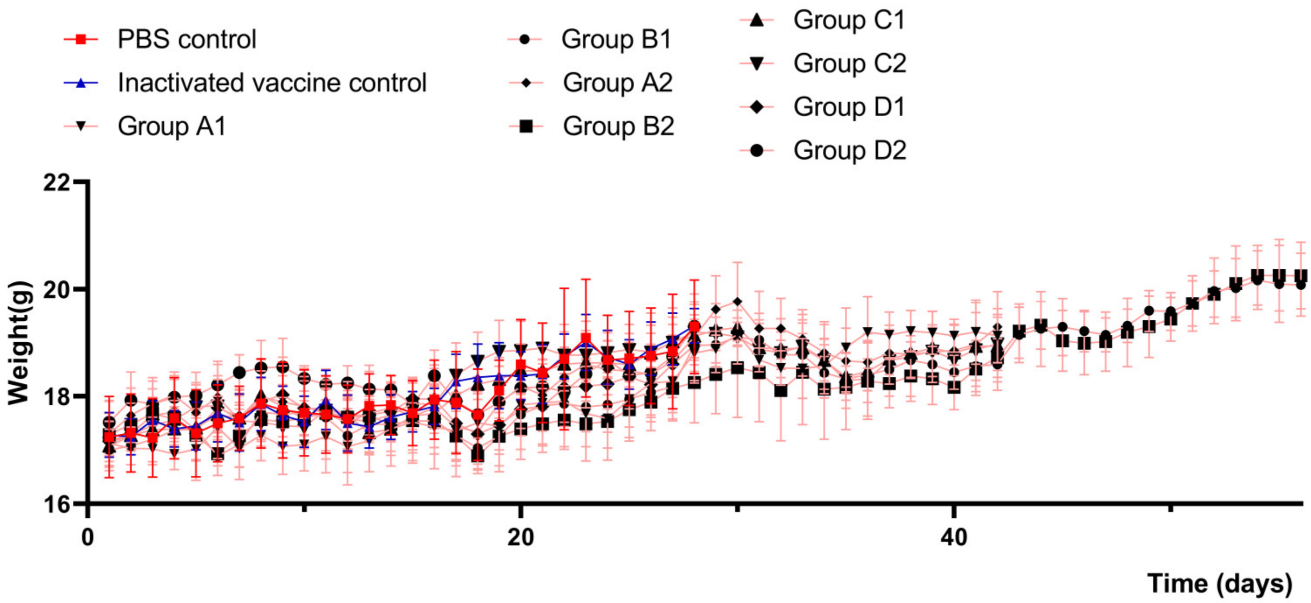

Figure S1. The growth of mice in different groups.
